# Advancing mental imagery research from an interdisciplinary sport science perspective: a commentary on Frank et al. (2023)

**DOI:** 10.1007/s00426-024-01942-z

**Published:** 2024-03-02

**Authors:** Howie J. Carson, Ray Bobrownicki

**Affiliations:** Human Performance Science Research Group, Institute for Sport, Physical Education and Health Sciences, Moray House School of Education and Sport, The University of Edinburgh, 4.19 St. Leonard’s Land, Holyrood Road, Edinburgh, EH8 8AQ UK

## Abstract

Frank et al.’s (2023) perceptual–cognitive scaffold meaningfully extends the cognitive action architecture approach and we support this interdisciplinary advancement. However, there are theoretical and applied aspects that could be further developed within this research to maximise practical impact across domains such as sport. In particular, there is a need to consider how these mechanisms (1) might critically inform or relate to other prominent theories within sport (e.g., constrained action hypothesis and ecological approaches) and, (2) reflect the real-world challenges experienced by athletes. With these ideas in mind, this commentary aims to stimulate discussion and enhance the translational application of Frank et al.’s research.

Although previous theories have incorporated the role of sensory information as components of movement planning (e.g., Ahsen, 1984; Lang, 1979; Lang et al., 1980), Frank et al. have posited that sensory-feedforward mechanisms are separate from motor representations used during action execution. The proposed perceptual–cognitive scaffold offers a meaningful and interdisciplinary extension of the most recent cognitive action architecture approach with potential implications across several domains. One such domain is sport with both applied and theoretical aspects of the scaffold that could be further explored to maximise practical impact. In this commentary, we (1) critically consider how this informs or relates to other prominent theories within sport (e.g., constrained action hypothesis and ecological approaches) and (2) reflect on real-world challenges of athletes. Accordingly, we aim to stimulate discussion and inform the translational application of Frank et al.’s research.

## Theoretical considerations

With theoretical advancements and elaboration, it is important to assess how new ideas align with existing concepts, models, and theories (see Fisher & Aguinis, 2017 for discussion on cognate areas of psychology). For us, the perceptual–cognitive scaffold has several theoretical implications to consider within the sporting literature.

First, Frank et al.’s conceptualisations of the scaffold and of perceptual effects during imagery, which emphasise the internal movement experience, contrasts and challenges the popular recommendations of Wulf and colleagues for a focus towards perceived *external* movement effects. Indeed, where Frank et al. acknowledged that “kinaesthetic aspects, feel of the movement, and bodily effects of the action” are relevant for imagery practice, Wulf and colleagues are resolute that such foci are detrimental to movement self-organisation and automaticity (Wulf, 2016; Wulf et al., 2001). Even with Wulf’s absolutist perspective receiving vigorous scholarly challenge (Collins et al., 2016; McKay et al., 2023; Robazza et al., 2016; Toner & Moran, 2015), it will be important to reconcile how the internal scaffolding process fits within these common views. In addition, there is a documented need to dynamically shift attention within performance contexts as part of motor planning. For example, a shift from an external focus of attention to an internal state of intention (i.e., active memory retrieval) is demonstrated by progressive visual disengagement in the moments prior to action execution in golf (Collins et al., 2023) and pistol shooting (Loze et al., 2001). This suggests that an external focus of attention may inform the perceptual–cognitive scaffold for these types of tasks, but that dysfunctional aspects associated with this shift (e.g., distraction) could impact on cognitive resources available during this latter movement planning phase. Accordingly, in reconciling these internal and external processes, there is a need to consider how the scaffold integrates with broader aspects of the performance environment and preparation process.

Second, the tentative and evolving nature of the scaffold as a set of performance expectations aligns with predictive-processing research, which has diverted from traditional conceptualisations of motor commands toward probabilistic models (Parr et al., 2022). As one challenge to performance, movements frequently require updating as circumstances unfold in action (e.g., adapting tactical play mid-rally in tennis while simultaneously executing a range of shots). Indeed, as Frank et al. stated, “the resulting scaffold may not be the final and most appropriate one for a given action, but helps the learner as an estimate, a frame or a model, for future (overt) action control”. Therefore, not only might the initial process of motor activation represent an important concern, but also the ongoing need for flexibility. In the sport literature, there is also considerable debate between the role of indirect and direct (e.g., derived from ecological psychology) perception in the execution of such contextually situated and adaptive motor skills (Bobrownicki et al., 2023). Whether indirect or direct mechanisms might best explain this process, the debate within sport raises the need for the perceptual–cognitive scaffolding approach to address the adaptive demands of athletes.

## Applied considerations

Within sport, there are several applied considerations for maximising relevance and translation of the perceptual–cognitive scaffold to address real-world challenges. First, athletes will invariably observe the performances of others and, in turn, receive perceptual information about the to-be-performed task and movements. As such, the perceptual–cognitive scaffold will interact with biopsychosocial factors that can influence, for instance, the level of learner engagement (e.g., due to social relatedness to model performers) and consequent effects (e.g., increased confidence). Frank et al. highlighted that:

For complex tasks with high motor components that require coordination between body parts or new coordination pattern…, the perceptual-cognitive scaffolding does not necessarily lead to changes in overt behavior, and leads to motor learning only if a link between anticipated effects and the related coordination pattern exists.

A crucial issue within sport is, therefore, knowing *whom* to watch, *what* to watch and *why* in order to avoid misalignment between the scaffold and the to-be-performed task *for each learner*. Key to the success of the perceptual–cognitive scaffold will be the coherence between the perceived and *actual* motor strategy. For complex, whole-body movements, such as sports skills where there are large amounts of inter-individual variability between athletes (e.g., Glazier & Mehdizadeh, 2019), it is important that the stimulus is appropriate for the learner across a range of factors (Ste-Marie et al., 2012). Indeed, research in sport is increasingly advocating an interactive understanding of biopsychosocial processes that are relevant in this context (Taylor et al., 2018). For example, mismatches between what the learner imagines the perceptual effect to be and their actual technical capability may result in poor performance, confusion, injury, demotivation, and hindered development (e.g., “I *think* I’m swinging it like Tiger Woods, but why am I not improving?”). So, for practitioners, when providing observational stimuli to inform perceptual–cognitive scaffolding, the model characteristics relating to physical similarity and action intention require careful consideration. Exemplar guidelines within the sport and exercise domain include consideration of the movement timing, physical arousal state, outcome, imagery ability and the cognitive developmental stage of the learner (MacIntyre et al., 2013). One such observational model that has received comparatively limited attention within sport research includes the best-self model proposed by Carson et al. (2014) that has the potential to provide both accurate perceptual information and motivational impact toward skill learning and refinement. Recent research has also focused on biopsychosocial benefits of simultaneously combining motor imagery when observing a recorded model performance (see Scott et al., 2022, for a detailed overview). We welcome further research on the perceptual–cognitive scaffold relating to complex, whole-body movements and associated challenges and demands.

Another important applied consideration relates to the difficulty or challenge of the task, as this will influence engagement with imagery and the resultant accuracy of any predicted perceptual effects. In this regard, practice tasks that are too easy may not require sufficient engagement for this conscious perceptual process, whereas tasks that are too difficult may prevent or frustrate the development of realistic predictions regarding movement effects. Indeed, the development of concurrent metacognitive processes (e.g., monitoring) that underpin one’s ability to adapt the perceptual–cognitive scaffold may enhance motor execution in response to differing situational demands (e.g., competitive pressure; Carson et al., 2020). In considering this, the practical application of the challenge point hypothesis (Guadagnoli & Lee, 2004; Hodges & Lohse, 2022) might offer a useful framework for informing imagery engagement and, in turn, the movement representation’s establishment within memory (Carson & Collins, 2016). Such consideration of physical and mental practice within motor control has the potential to inform more meaningful research designs for translational impact.

## Conclusion

This commentary welcomes the advancement of research relating to cognitive theory in motor learning that has relevance across domains. We hope that our distinct perspective from sport offers worthwhile and valuable feedback to consider the ideas proposed by Frank et al. and inform the development of theory and research. It has long been stated, but not always appreciated, that knowledge generation benefits from discussion and debate across stakeholders relevant to and informed by the research (Christina, 1987).

## Data Availability

Not Applicable.
